# Supplementary material for: COVID-19 Vaccine Hesitancy and Its Associated Factors in Japan
Source: Vaccines (Basel). 2021 Jun 17;9(6):662. doi: 10.3390/vaccines9060662 (PMC8234307; doi:10.3390/vaccines9060662)
Supplement: Supplementary file 1 [file vaccines-09-00662-s001.zip › vaccines-1212750-supplementary.pdf]

Supplementary Material

Title: COVID-19 vaccine hesitancy and associated factors in Japan

List:

Supplementary Methods S1-Statistical analysis

Table S1. Distribution of COVID-19 case numbers by prefecture.

Table S2. Participants’ baseline characteristics for analyses (before propensity score weighting).

Table S3. Proportion of COVID-19 vaccine hesitancy, stratified by age and sex.

Table S4. Reasons for getting vaccinated against the COVID-19 and not getting vaccinated, stratified by age.

Table S5. Logistic regression models for odds ratio of COVID-19 vaccine hesitancy, stratified by age.

Supplementary S1. Management of data quality.

Supplementary Methods S1- Statistical analysis

Selectivity of Internet-based samples were corrected for by the use of a population-based sample that was representative of the Japanese population from the Comprehensive Survey of Living Conditions of People on Health and Welfare (CSLCPHW). This survey is conducted every 3 years in the first week of June, investigating living conditions and collecting basic data required by the Japanese Ministry of Health, Labour and Welfare (MHLW) for planning and management. It covers households and household members nationwide, and requires participant responses to a detailed self-reported questionnaire. Of 940,000 sampling districts included in population censuses in 2010, random sampling of 5410 districts was performed in 2016 for CSLCPHW data collection. In each district, all household members were asked to respond to a questionnaire on living conditions and health. Within the 5410 sampling districts, there 289,470 households, of which 224,208 responded to the questionnaire (response rate, 77.5%). The MHLW permitted use of these data in the present study.

The data from both the present Internet survey and the CSLCPHW were combined and input into a logistic regression model, along with each of the abovementioned covariates to estimate the probability of "being a respondent in an Internet survey" (i.e., propensity score [PS]). We stratified by sex and age group (sex × age groups = 14 strata), and calculated the PS separately for each stratum [1, 2]. Data from the 2016 CSLCPHW were used because the 2019 CSLCPHW was not yet available at the time of analysis.

A multivariable logistic regression model was used to calculate the PS in each stratum (age and sex groups). Variables available in both of the surveys were used in the models. For respondents aged 20-79 years, we adjusted for area (residence), marital status, education, homeownership (household), self-rated health, and smoking status. For respondents aged 15-19 years, we adjusted for area, education, homeownership (household), and self-rated health, but omitted marital and smoking status because this age group was too young to yield a different distribution of marital status and the Ministry stopped asking teenagers about their smoking status from 2013.

References

1. Tabuchi, T.; Shinozaki, T.; Kunugita, N.; Nakamura, M.; Tsuji, I. Study Profile: The Japan "Society and New Tobacco" Internet Survey (JASTIS): A Longitudinal Internet Cohort Study of Heat-Not-Burn Tobacco Products, Electronic Cigarettes, and Conventional Tobacco Products in Japan. Journal of epidemiology 2019, 29, 444-450, doi:10.2188/jea.JE20180116.
2. Tabuchi T, Shinozaki T, Kunugita N et al. Study Profile: The Japan "Society and New Tobacco" Internet Survey (JASTIS): A Longitudinal Internet Cohort Study of Heat-Not-Burn Tobacco Products, Electronic Cigarettes, and Conventional Tobacco Products in Japan. J Epidemiol 2019; 29: 444-450.

Table S1. Distribution of COVID-19 case numbers by prefecture.

	Prefecture-level cumulative number of COVID-19 cases per 1,000,000 population until Feb 7, 2021<br>Mean (min-max) case	
	Lower	Higher
Number of COVID-19 cases by prefecture	1472 (278-3135)	4751 (3270-7520)
Prefectures	Akita, Tottori, Shimane, Iwate, Niigata, Yamagata, Tokushima, Aomori, Fukui, Kagawa, Ehime, Toyama, Yamaguchi, Fukushima, Kagoshima, Oita, Yamanashi, Nagano, Nagasaki, Wakayama, Saga, Kochi, Okayama, Shizuoka, Mie, Ishikawa, Miyazaki, Shiga, Hiroshima, Miyazaki, Ibaraki, Kumamoto, Tochigi, Gunma, Gifu, Nara, Hyogo,	Aichi, Fukuoka, Kyoto, Hokkaido, Saitama, Chiba, Kanagawa,<br>Osaka, Okinawa, Tokyo

Values throughout Japan until Feb 7, 2021 equal 3203 cases per 1,000,000 population.

Table S2. Participants' baseline characteristics included in analyses (before propensity score weighting).

	**Total (N = 23,142)**	
	**N**	**%**
Sex, Female	11,376	49.2
Age group (years)		
Younger (15-39)	6686	28.9
Middle-aged (40-64)	10,554	45.6
Older (65-79)	5903	25.5
Income (million JPY/year)		
less than 1	841	3.6
1 to less than 6	10,699	46.2
6 to less than 120	5433	23.5
120 or more	952	4.1
No response/unknown	5217	22.5
Marital status		
Never married	5697	24.6
Married	15,262	65.9
Widowed/divorced	2183	9.4
Living alone, yes	3076	13.3
Occupation		
Health care professional	877	3.8
Essential worker in the food industry	3381	14.6
Other occupation	9878	42.7
Unemployed	9006	38.9
Educational level		
Junior high school graduate	4114	17.8
High school graduate	9739	42.1
Two-year college graduate	3366	14.5
Bachelor's degree	4315	18.6
Master's or doctoral degree	1608	7.0
Use of combustible cigarettes or HTPs		
Neither	19,024	82.2
Only HTPs	2286	9.9
Only combustible cigarettes	915	4.0
Dual use	917	4.0
Alcohol use		
Never	7269	31.4
Ever	7717	33.3
Current	8156	35.2
Comorbidity (present)		
Hypertension	4359	18.8
Diabetes mellitus	1628	7.0
Asthma or COPD	1031	4.5
Cardiovascular disease	310	1.3
Cerebrovascular disease	260	1.1
Cancer	442	1.9
Chronic pain	2330	10.1
Psychiatric disorders	1463	6.3
Personal history of COVID-19 infection	400	1.7
Fear of COVID-19-induced death	7884	34.1
Perceived likelihood of getting infected with COVID-19 themself	3197	13.8
Distrust toward the government	16,962	73.3
Distrust toward government policy on COVID-19	15,724	67.9
The thought of embarrassment of getting infected with COVID-19	5463	23.6

Severe psychological distress, yes	2741	11.8
Living in a prefecture with a high proportion of COVID-19 cases	7730	33.4

Abbreviations: JPY, Japanese Yen; HTP, heated tobacco product; COPD, chronic obstructive pulmonary disease; COVID-19, coronavirus disease 2019.

Table S3. Proportion of COVID-19 vaccine hesitancy, stratified by age and sex.

	COVID-19 Vaccine Intention		
	Intend		Hesitant
	"I want to be vaccinated"	"I want to be vaccinated after seeing how it goes"	"I don't want to be vaccinated"
All	35.9	52.8	11.3
Male respondents	41.7	48.1	10.2
Younger (15-39 years)	31.0	54.8	14.2
Middle-aged (40-64 years)	38.1	51.3	10.6
Older (65-79 years)	36.5	18.3	4.8
Female respondents	29.9	57.6	12.5
Younger (15-39 years)	18.8	65.6	15.6
Middle-aged (40-64 years)	27.1	59.8	13.2
Older (65-79 years)	47.6	44.7	7.7

Analyses were weighted to adjust for differences between Internet survey respondents and nationally representative samples. Abbreviation: COVID-19, coronavirus disease 2019.

Table S4. Reasons for getting vaccinated against COVID-19, or not getting vaccinated, stratified by age.

**(A) Among younger respondents aged 15-39 years.**		
	**COVID-19 Vaccine Intention**	
	**Intend, %**	**Hesitant, %**
Reasons for getting vaccinated, %		
It was recommended by a family member or friend	4.4	
It was recommended by SNS or the media	3.7	
I'm worried about getting infected with COVID-19	24.3	
I think I have a high risk of becoming seriously ill	5.0	
I am a medical worker	5.4	
I don't want to infect my family or other people around me	25.0	
I think it is necessary for society to be vaccinated	21.7	
I can get it for free	15.3	
Reasons for not getting vaccinated, %		
I don't have time to go get vaccinated		12.4
I'm worried about adverse reactions		68.1
I don't think it is very effective		19.1
I don't think I will get infected.		9.6
I think I have a low risk of getting seriously ill		10.4
I was previously infected with COVID-19		0.3
I have received the COVID-19 vaccine		0.8
It was recommended by a family member or friend		2.2
It was recommend by SNS or the media		4.7

**(B) Among middle-aged respondents aged 40-64 years.**		
	**COVID-19 Vaccine Intention**	
	**Intend, %**	**Hesitant, %**
Reasons for getting vaccinated, %		
It was recommended by a family member or friend	2.0	
It was recommended by SNS or the media	1.3	
I'm worried about getting infected with the COVID-19	30.0	
I think I have a high risk of becoming seriously ill	12.6	
I am a medical worker	3.7	
I don't want to infect my family or other people around me	32.7	
I think it is necessary for society to be vaccinated	29.5	
I can get it for free	18.0	
Reasons for not getting vaccinated, %		
I don't have time to go get vaccinated		7.7
I'm worried about adverse reactions		75.8
I don't think it is very effective		19.9
I don't think I will get infected.		6.8
I think I have a low risk of getting seriously ill		6.4
I was previously infected with COVID-19		0.4
I have already received the COVID-19 vaccine		0.2
It was recommended by a family member or friend		0.5
It was recommended by SNS or the media		1.9

**(C) Among older respondents aged 65-79 years.**		
	**COVID-19 Vaccine Intention**	
	**Intend, %**	**Hesitant, %**
Reasons for getting vaccinated, %		
It was recommended by a family member or friend	2.4	-
It was recommended by SNS or the media	1.1	-
I'm worried about getting infected with the COVID-19	47.6	-
I think I have a high risk of becoming seriously ill	37.2	-
I am a medical worker	2.0	-

I don't want to infect my family or other people around me	43.1	-
I think it is necessary for society to be vaccinated	45.7	-
I can get it for free	22.5	-
Reasons for not getting vaccinated, %		
I don't have time to go get vaccinated	-	2.5
I'm worried about adverse reactions	-	83.3
I don't think it is very effective	-	18.6
I don't think I will get infected	-	5.9
I think I have a low risk of getting seriously ill	-	3.6
I was previously infected with COVID-19	-	1.1
I have already received the COVID-19 vaccine	-	0.0
It was recommended by a family member or friend	-	1.4
It was recommended by SNS or the media	-	0.3

Analyses were weighted to adjust for differences between Internet survey respondents in the present study and nationally representative samples. Abbreviations: COVID-19, coronavirus disease 2019; SNS, social network services.

Table S5. Logistic regression models for odds ratio of COVID-19 vaccine hesitancy, stratified by age.

**(A) Among younger respondents aged 15-39 years.**			
	**aOR**	**95% CI**	**p-value**
Sex, female	1.04	(0.89-1.21)	0.61
Income (million JPY/year)			
less than 1	1.39	(1.03-1.87)	0.03
1 to less than 6	ref	-	-
6 to less than 120	0.82	(0.68-1.00)	0.04
120 or more	0.65	(0.41-1.01)	0.06
No response/unknown	1.11	(0.93-1.33)	0.27
Marital status			
Never married	ref	-	-
Married	0.89	(0.75-1.05)	0.16
Widowed/divorced	1.39	(0.94-2.06)	0.10
Living alone, yes	1.46	(1.18-1.81)	<0.01
Occupation			
Health care professional	0.96	(0.70-1.31)	0.79
Essential worker in the food industry	1.02	(0.83-1.24)	0.88
Other occupation	ref	-	-
Unemployed	0.94	(0.79-1.11)	0.46
Educational level			
Junior high school graduate	1.16	(0.93-1.44)	0.20
High school graduate	0.95	(0.78-1.16)	0.61
Two-year college graduate	1.34	(1.07-1.69)	0.01
Bachelor's degree	ref	-	-
Master's or doctoral degree	1.01	(0.61-1.67)	0.96
Use of combustible cigarettes or HTPs			
Neither	ref	-	-
Only HTPs	0.62	(0.44-0.87)	0.01
Only combustible cigarettes	1.83	(1.36-2.46)	<0.0001
Dual use	0.59	(0.39-0.90)	0.01
Alcohol use			
Never	ref	-	-
Ever	0.56	(0.48-0.66)	<0.0001
Current	0.62	(0.50-0.75)	<0.0001
Comorbidity (present)			
Hypertension	0.53	(0.31-0.92)	0.02
Diabetes mellitus	0.46	(0.24-0.88)	0.02
Asthma or COPD	1.75	(1.23-2.51)	<0.01
Cardiovascular disease	1.37	(0.58-3.21)	0.47
Cerebrovascular disease	1.17	(0.51-2.69)	0.71
Cancer	1.24	(0.58-2.65)	0.57
Chronic pain	0.63	(0.45-0.89)	0.01
Psychiatric disorders	0.87	(0.66-1.16)	0.34
Personal history of COVID-19 infection	1.37	(0.95-1.97)	0.09
Fear of COVID-19-induced death	0.60	(0.50-0.71)	<0.0001
Perceived likelihood of getting infected with COVID-19 themself	0.94	(0.76-1.16)	0.56
Distrust toward the government	1.48	(1.19-1.84)	<0.01
Distrust toward government policy on COVID-19	1.15	(0.95-1.40)	0.14
The thought of embarrassment of getting infected with COVID-19	0.97	(0.82-1.14)	0.70
Severe psychological distress, yes	1.30	(1.09-1.56)	0.00
Living in a prefecture with a high proportion of COVID-19 cases	0.94	(0.81-1.10)	0.44
**(B) Among middle-aged respondents aged 40-64 years.**			

	aOR	95% CI	p-value
Sex, female	1.11	(0.96-1.28)	0.15
Income (million JPY/year)			
less than 1	1.38	(1.03-1.84)	0.03
1 to less than 6	ref	-	-
6 to less than 120	0.81	(0.69-0.96)	0.01
120 or more	1.02	(0.77-1.36)	0.88
No response/unknown	1.19	(1.02-1.39)	0.03
Marital status			
Never married	ref	-	-
Married	0.61	(0.51-0.72)	<.0001
Widowed/divorced	0.79	(0.63-0.99)	0.04
Living alone, yes	1.25	(1.03-1.53)	0.03
Occupation			
Health care professional	0.61	(0.42-0.89)	0.01
Essential worker in the food industry	0.81	(0.68-0.97)	0.02
Other occupation	ref	-	-
Unemployed	1.04	(0.89-1.21)	0.66
Educational level			
Junior high school graduate	1.02	(0.82-1.27)	0.86
High school graduate	1.14	(0.96-1.37)	0.14
Two-year college graduate	1.20	(0.97-1.48)	0.09
Bachelor's degree	ref	-	-
Master's or doctoral degree	0.92	(0.55-1.56)	0.76
Use of combustible cigarettes or HTPs			
Neither	ref	-	-
Only HTPs	0.95	(0.78-1.15)	0.57
Only combustible cigarettes	0.69	(0.50-0.97)	0.03
Dual use	0.77	(0.56-1.06)	0.10
Alcohol use			
Never	ref	-	-
Ever	0.72	(0.62-0.84)	<.0001
Current	0.70	(0.60-0.81)	<.0001
Comorbidity (present)			
Hypertension	0.99	(0.84-1.16)	0.90
Diabetes mellitus	0.53	(0.39-0.73)	<.0001
Asthma or COPD	0.95	(0.67-1.34)	0.76
Cardiovascular disease	0.82	(0.34-2.00)	0.67
Cerebrovascular disease	1.34	(0.74-2.43)	0.34
Cancer	1.44	(0.90-2.30)	0.13
Chronic pain	1.35	(1.11-1.63)	<0.01
Psychiatric disorder	0.54	(0.41-0.72)	<.0001
Personal history of COVID-19 infection	1.38	(0.78-2.41)	0.27
Fear of COVID-19-induced death	0.55	(0.47-0.64)	<.0001
Perceived likelihood of getting infected with COVID-19 themself	1.15	(0.94-1.41)	0.17
Distrust toward the government	1.06	(0.86-1.30)	0.59
Distrust toward government policy on COVID-19	1.31	(1.08-1.58)	0.01
The thought of embarrassment of getting infected with COVID-19	0.87	(0.75-1.02)	0.08
Severe psychological distress, yes	1.58	(1.32-1.90)	<0.0001
Living in a prefecture with a high proportion of COVID-19 cases	0.98	(0.86-1.12)	0.81

(C) Among older respondents aged 65-79 years.

	aOR	95% CI	p-value
Sex, female	2.24	(1.68-3.00)	<0.0001
Income (million JPY/year)			

less than 1	6.40	(4.06-10.09)	<0.0001
1 to less than 6	Ref	-	-
6 to less than 120	0.55	(0.34-0.90)	0.02
120 or more	0.49	(0.12-2.10)	0.34
No response/unknown	0.37	(0.26-0.54)	<0.0001
Marital status			
Never married	ref	-	-
Married	1.43	(0.82-2.49)	0.21
Widowed/divorced	0.43	(0.25-0.73)	<0.01
Living alone, yes	2.00	(1.24-3.22)	<0.01
Occupation			
Health care professional	0.16	(0.01-2.48)	0.19
Essential worker in the food industry	0.48	(0.25-0.94)	0.03
Other occupation	ref	-	-
Unemployed	0.50	(0.39-0.66)	<0.0001
Educational level			
Junior high school graduate	2.11	(1.36-3.27)	<0.01
High school graduate	1.56	(1.06-2.31)	0.03
Two-year college graduate	1.51	(0.87-2.62)	0.15
Bachelor's degree	ref	-	-
Master's or doctoral degree	1.11	(0.70-1.77)	0.66
Use of combustible cigarettes or HTPs			
Neither	ref	-	-
Only HTPs	1.51	(1.04-2.19)	0.03
Only combustible cigarettes	3.96	(1.88-8.34)	<0.01
Dual use	0.33	(0.07-1.56)	0.16
Alcohol use			
Never	ref	-	-
Ever	0.77	(0.57-1.03)	0.07
Current	0.59	(0.43-0.80)	<0.01
Comorbidity (present)			
Hypertension	0.81	(0.61-1.07)	0.14
Diabetes mellitus	0.66	(0.43-0.99)	0.04
Asthma or COPD	0.56	(0.28-1.08)	0.08
Cardiovascular disease	4.66	(2.56-8.49)	<0.0001
Cerebrovascular disease	0.94	(0.29-3.09)	0.92
Cancer	0.24	(0.09-0.65)	0.01
Chronic pain	0.66	(0.43-1.00)	0.05
Psychiatric disorder	0.36	(0.14-0.93)	0.03
Personal history of COVID-19 infection	5.96	(1.48-23.94)	0.01
Fear of COVID-19-induced death	0.42	(0.31-0.55)	<0.0001
Perceived likelihood of getting infected with COVID-19 themself	0.71	(0.46-1.10)	0.13
Distrust toward the government	1.61	(1.02-2.52)	0.04
Distrust toward government policy on COVID-19	1.24	(0.82-1.87)	0.32
The thought of embarrassment of getting infected with COVID-19	2.40	(1.84-3.12)	<0.0001
Severe psychological distress, yes	2.78	(1.46-5.29)	<0.01
Living in a prefecture with a high proportion of COVID-19 cases	1.18	(0.92-1.52)	0.19

Analyses were weighted to adjust for differences between Internet survey respondents in the present study and nationally representative samples. Abbreviations: JPY, Japanese Yen; HTP, heated tobacco product; COPD, chronic obstructive pulmonary disease; COVID-19, coronavirus disease 2019; aOR, adjusted odds ratios; CI, confidence interval.

Supplementary S1. Management of data quality

We designed exclusion criteria to confirm data quality. The criteria were as follows:

(A) Attention check

We excluded respondents who answered incorrectly to the following question:
Please choose the second from the bottom of the following options.

A

B

C

D

E

*The correct answer is D.

(B1) straight-lining

We excluded respondents who answered "almost every day" or "occasionally" (as opposed to "habitually in the past," "several times in the past," or "never") to all nine questionnaires regarding substance use:

(1) alcohol, (2) sleeping pills/anti-anxiety drugs, (3) prescribed narcotics for cancer pain, (4) prescribed narcotics for non-cancer pain, (5) non-prescribed narcotics, (6) inhalation of organic solvents such as paint thinner or toluene, (7) illegal herbs/magic mushrooms, (8) cannabis (marijuana), and (9) methamphetamine/cocaine/heroin.

(B2) straight-lining

We excluded respondents who answered "currently have this condition and receiving treatment" or "currently have this condition but not receiving treatment" (as opposed to "never in the past" or "not now, but existed in the past") to all 16 questions regarding the following comorbidities:

(1) hypertension, (2) diabetes, (3) asthma, (4) bronchitis/pneumonia, (5) atopic dermatitis, (6) periodontal disease, (7) caries, (8) otitis media, (9) angina pectoris, (10) myocardial infarction, (11) stroke, (12) chronic obstructive pulmonary disease, (13) cancer/malignant tumor, (14) chronic pain, (15) depression, and (16) mental disorder other than depression.”
